# The effect of pre-pregnancy hair dye exposure on infant birth weight: a nested case-control study

**DOI:** 10.1186/s12884-018-1782-5

**Published:** 2018-05-09

**Authors:** Chao Jiang, Qingzhi Hou, Yaling Huang, Juan Ye, Xiaolian Qin, Yu Zhang, Wen Meng, Qiuyan Wang, Yonghua Jiang, Haiying Zhang, Mujun Li, Zengnan Mo, Xiaobo Yang

**Affiliations:** 1 Center for Genomic and Personalized Medicine, Guangxi Medical University, Nanning, Guangxi Zhuang Autonomous Region China; 2 Guangxi key Laboratory for Genomic and Personalized Medicine, Nanning, Guangxi Zhuang Autonomous Region China; 3 Guangxi Collaborative Innovation Center for Genomic and Personalized Medicine, Nanning, Guangxi Zhuang Autonomous Region China; 4 Guangxi Key Laboratory of Colleges and Universities, Nanning, Guangxi Zhuang Autonomous Region China; 5 Department of Occupational and Environmental Medicine School of Public Health, Guangxi Medical University, 22 Shuangyong Rd, Nanning, 530021 Guangxi China; 6 Department of Antenatal care, the Maternal & Child Health Hospital of Yulin, Yulin, Guangxi China; 7 Department of Gynecology and Obstetrics, the Maternal & Child Health Hospital of Liuzhou, Liuzhou, Guangxi China; 8 Department of Medical Services Section, the Maternal & Child Health Hospital of Guigang, Guigang, Guangxi China; 9 Department of Reproductive Center, First Affiliated Hospital of Guangxi Medical University, Nanning, Guangxi China

**Keywords:** Hair dye use, Pre-pregnancy BMI, Irregular menstruation, Low birth weight infants, Macrosomia

## Abstract

**Background:**

Limited evidences were reported about the risk of pre-pregnancy hair dye use or irregular menstruation with abnormal birth weight during pregnancy, and their joint effects were also unknown. The aim of our study was to explore whether the pre-pregnancy exposure of hair dye and irregular menstruation were associated with the risk of abnormal birth weight.

**Methods:**

We conducted a nested case-control study from a prospective cohort of 6203 pregnant women. Low birth weight study included 315 mother-infant pairs (105 LBW cases and 210 matched controls), and macrosomia study included 381 mother-infant pairs (127 macrosomia cases and 254 matched controls). Meanwhile, lifestyle information including hair dying custom and menstrual history were collected by face-to-face questionnaires and birth outcomes were extracted from the medical records. The logistic regressions models were used to analyze the join effect of irregular menstruation and hair dye use.

**Results:**

Pre-pregnancy hair dye use was associated with increased risk of LBW (adjusted OR = 1.71, 95% CI: 1.01–2.92, *P* = 0.048). Irregular menstruation had high risk of LBW (adjusted OR = 2.79, 95% CI: 1.53–5.09, *P* = 0.001) and macrosomia (adjusted OR = 1.93, 95% CI: 1.09–3.44, *P* = 0.023). Additionally, in the LBW study, women who used hair dye with pre-pregnancy BMI < 18.5 kg/m^2^ had higher OR than those with only one risk factor (3.07 vs 2.53, *P*_trend_ = 0.015), and women with both hair dye use and irregular menstruation also had higher risk than those with only one factor (4.53 vs 2.07, *P*_trend_ = 0.05). Moreover, in macrosomia study, women with irregular menstruation and pre-pregnancy BMI ≥ 24 kg/m^2^ had higher risk than those with one factor (13.31 vs 2.09, *P*_trend_ = 0.001).

**Conclusion:**

Our study showed that either pre-pregnancy hair dye use or irregular menstruation was associated with abnormal birth weight, especially, their joint effects could furthermore increase the risk of low birth weight infants when these two factors existed simultaneously.

## Background

Abnormal birth weight is an important issue which could reflect fetal growth and maternal health, including low birth weight (LBW) infant (live infant weight < 2, 500 g at birth) and macrosomia (live infant weight > 4, 000 g at birth) [1]. Low birth weight infants comprise 7.6% of all live-born infants in United States [2, 3] and 5.87% in China [4]. Additionally, macrosomia rate is 4 to 20% in Western countries and 6.5 to 11.67% in China [5, 6].The abnormal birth weight might have effects on fetus or children sub-health [7–9], and even were associated with diseases such as obesity, cancer, cardiovascular diseases, hypertension and malnutrition [5, 10, 11]. Thus, newborn birth weight is regarded as a significant problem of public health [12].

The etiology of abnormal birth weight is not entirely clear now, previous studies reported maternal pre-pregnancy body mass index (BMI), life style, dietary and infection during pregnancy, et al. may have effects on abnormal birth weight [7–9, 13, 14]. As society develops, the life style of women have drastically changed, fast-paced work and irregular living habits may affect internal secretion of ovary which could affect pregnancy outcomes. Because hair dye use and irregular menstruation is increasing and becoming common during women [15, 16], we hypothesis certain chemicals which present in hair dye and irregular menstruation may have effect on abnormal birth weight infant. The products of hair dye contain large parts of chemical substances, such as aromatic amines, organic solvents, phthalates, formaldehyde, et al. Some studies indicated that the reproductive toxicity of chemical substances had adverse effect on birth outcomes in animal experiments [17, 18]. Previous epidemiological studies showed that occupational hair-dressers and cosmetologist had reproductive risks on abnormal pregnancy outcomes such as LBW, preterm birth, abortion, preeclampsia [19–22]. In general population, it is also common for women to have irregular menstruation, which reaches up to 30% between the age 18 and 40 [16], and those women were reported to have high risk of pregnant complications, such as low birth weight [23], preterm birth, preeclampsia, gestational diabetes mellitus,et al. [23–25]. However, few study reported the risk of hair dye use for those female customers, and simultaneously targeted the women with irregular menstruation and the hair dye use pre-pregnancy on the risk of abnormal birth weight infant.

Our present study used the nested case-control design including 4,978 pregnant women in Guangxi, China, and investigated associations between pre-pregnancy hair dye use, irregular menstruation and the risk of abnormal birth weight.

## Methods

### Study design and population

The nested case-control study is based on an ongoing collaborate prospective of Guangxi Birth Cohort Study (GBCS), which contains seven Maternal & Child Health Hospitals and aims to investigate the effects of genetic and environmental factors on pregnancy outcomes. The inclusion criteria of GBCS required the volunteers to be 18–45 years old, born in and live in the study areas and have comprehensive ability to complete a face-to-face interview questionnaire. A total of 6, 203 pregnant women were recruited during prenatal examination at those hospitals from July to September in 2015. In the end, 5, 541 pregnancies were followed-up after they completed the delivery.

In the nested case-control study, the exclusion criteria were: (1) Preterm delivery (< 37 gestational weeks), multiple birth, unknown infant gender, abortion, stillbirth and birth defect. (2) Women who used birth control pills or contraceptive injection less than few months prior to pregnancy or did any fertility treatment were excluded. After exclusions, 4, 978 single live full-term infant-mother pairs were selected for the final study, contained 105 LBW infants and 127 macrosomia. The selection of the controls was as follows: (1) Mothers delivered a singleton live full-term infant with normal birth weight. (2) For each case, two controls were randomly selected from the normal birth-weight subjects based on the matching variables as fetal gender, approximate gestational age at conception (within 1-week interval), maternal age (within 1-age interval) and recruitment area According to the matching constraints, separately 210 normal birth-weight births were matched for low birth weight cases and 254 controls were matched for macrosomia cases.

### Data collection

The detailed questionnaire collected information, regarding demographic factors (education level, maternal age and pre-pregnancy height and weight by self-reported, et al.*..*), personal lifestyle (smoking, alcohol consumption and hair dye use history, et al), pregnancy history (gravidity and parity), pregnancy complications, medication history (pre-pregnancy use and during pregnancy use), physical activity, dietary, psychological factors and the menstrual history. Pregnant women were followed-up until delivery, and the information of pregnant women (age, parity, pregnant disease, and gravidity) and infants (gender, Apgar score of 5 min, weight, length, head and chest circumference) were obtained from the Guangxi Maternal and Child Health Information System.

Hair dye exposure was defined as women used hair dye before conception which was collected from questionnaires by self-reported. Irregular menstruation was defined as menstrual cycle persistently outside 21–45 days [20] by self-reported [13]. The neonatal outcome of this study was live infant birth weight which was calculated within 5 min after birth. < 2, 500 g is low birth weight, and macrosomia is > 4, 000 g at birth [1]. Gestational age was assessed by means of the date of the last menstrual period and fetal biometry in the first trimester ultrasound scan.

### Ethical approval

Study protocol data collection was approved by Medical Ethics Committee of First Affiliated Hospital of Guangxi Medical University (ID: 2015(028)). All patients consented to participate in the research, and written informed consent was obtained from each patient.

### Statistical analysis

Significant differences were assessed by t-test for continuous variables (means with 1 SD) or chi-square tests for categorical variables (frequency and percentage). Binary logistic regression analyses were used examine the risk of dichotomized exposures associated with abnormal birth weight. Crude and adjusted Odds ratios (ORs) were calculated and 95% confidence intervals (CIs) were given. The pre-pregnancy hair dye use and maternal irregular menstruation were analyzed as categorical variables. A *P* value < 0.05 in 2-tailed tests was considered statistically significant. Statistical analysis was performed using SPSS version 21.0 (IBM).

## Results

### General characteristic

The nested case-control study contained two sub-studies, which were low birth weight (LBW) study and macrosomia study, respectively. General characteristic of maternal and neonatal are presented in Tables 1 and 2.

**Table 1:** Baseline characteristics of two sub-study subjects

Characteristics	LBW group (*n* = 315)			Macrosomia group (*n* = 381)		
	LBW (%)	NBW (%)	*P* ^*a*^	Macrosomia (%)	NBW (%)	*P* ^*a*^
N	105	210		127	254	
Maternal age (years) [mean (SD)]	28 (4.7)	28.2 (4.9)	0.753	29.9 (5.2)	27.7 (4.8)	< 0.001
Pre-pregnant height (cm) [mean (SD)]	156.1 (4.3)	157.7 (4.7)	0.003	56.8 (8.6)	50 (7.6)	< 0.001
Pre-pregnant weight (kg) [mean (SD)]	47.8 (7.5)	51 (8.3)	0.001	158.4 (4.6)	157.3 (4.8)	0.047
Pre-pregnant BMI categories (kg /m ^2^)			0.073			< 0.001
< 18.5	40 (38.5)	56 (26.7)		8 (6.5)	78 (30.7)	
18.5–23.9	55 (52.9)	125 (59.5)		79 (63.7)	153 (60.2)	
≥ 24	9 (8.7)	29 (13.8)		37 (29.8)	23 (9.1)	
Age at menarche (years) [mean (SD)]	13.4 (1.5)	13.5 (1.5)	0.846	13.3 (2.1)	13.6 (1.7)	0.184
Gravidity history			0.212			0.073
First	44 (41.9)	67 (31.9)		28 (22.0)	71 (28.0)	
2–3	46 (43.8)	106 (50.5)		69 (54.3)	146 (57.5)	
> 3	15 (14.3)	37 (17.6)		30 (23.6)	37 (14.6)	
Parity history			0.145			0.017
First	37 (35.2)	92 (43.8)		71 (55.9)	109 (42.9)	
≥ 2	68 (64.8)	118 (56.2)		56 (44.1)	145 (57.1)	
Menstrual regularity			< 0.001			0.009
Yes	74 (70.5)	183 (87.1)		92 (72.4)	212 (83.8)	
Education level (years)			0.909			< 0.001
≤ 9	38 (36.2)	72 (34.3)		68 (53.5)	85 (33.5)	
10–12	32 (30.5)	63 (30.0)		30 (23.6)	72 (28.4)	
≥ 13	35 (33.3)	75 (35.7)		29 (22.8)	97 (39.2)	
Alcohol use pre-pregnancy			0.854			0.094
Yes	27 (25.7)	52 (24.8)		70 (27.6)	25 (19.7)	
Smoking status			0.213			0.554
Current / ex-smoker	3 (2.9)	1 (0.5)		2 (0.8)	0 (0)	
Second-hand smoking			0.657			0.257
Yes	16 (15.2)	28 (13.4)		26 (10.2)	18 (14.2)	
Progesterone use pre-pregnancy			0.282			0.857
Yes	1 (1.0)	8 (3.8)		11 (4.3)	5 (3.9)	
Progesterone use during pregnancy			0.375			1.000
Yes	26 (24.8)	62 (29.5)		38 (29.9)	76 (29.9)	

*Abbreviations*: *LBW* low birth weight, *NBW* new birth weight, *SD* standard deviation, % percentages estimated on valid data only, *BMI* body mass index is measured and defined as weight (kg) / height (m) ^2^

^a^T-tests and Chi-square tests were used to test differences in two sub-study subjects

**Table 2:** Selected lifestyle characteristics of two sub-study subjects

Exposure variables	LBW group (*n* = 315)			Macrosomia group (*n* = 381)		
	LBW (%)	NBW (%)	*P* ^a^	Macrosomia (%)	NBW (%)	*P* ^a^
N	105	210		127	254	
Nearby chemical factory			0.303			0.934
Yes	4 (3.8)	14 (6.7)		4 (3.2)	10 (3.9)	
Recent interior decoration			0.279			0.374
Yes	13 (12.5)	36 (17.2)		18 (14.2)	28 (11.0)	
Regularly cooking			0.806			0.094
Yes	76 (72.4)	154 (73.3)		97 (76.4)	173 (68.1)	
Chief fuel used for cooking			0.989			0.920
Pollution-type	11 (10.6)	22 (10.5)		15 (11.8)	29 (11.5)	
Ecological and clean-type	93 (89.4)	187 (89.5)		112 (88.2)	224 (88.5)	
Use of range hood			0.285			0.876
Yes	72 (68.6)	156 (74.3)		88 (69.3)	174 (68.5)	
Pets			0.584			0.731
Yes	29 (27.6)	52 (24.8)		28 (22.0)	60 (23.6)	
Radiation-proof clothes use during pregnancy			0.978			1.000
Yes	11 (10.6)	22 (10.5)		15(11.8)	30 (11.8)	
Lipstick use during pregnancy			0.717			0.054
Yes	6 (5.7)	10 (4.8)		2 (1.6)	15 (5.9)	
Hair dye use pre-pregnancy			0.047			0.175
Yes	75 (71.4)	126 (60.0)		75 (59.1)	168 (66.1)	

*Abbreviations*: *LBW* low birth weight, *NBW* new birth weight

^a^T-tests and Chi-square tests were used to test differences in two sub-study subjects

In the low birth weight study, compared to controls, there were lower pre-pregnant height and weight, and a higher proportion of irregular menstruation (29.5% vs 12.9%), all *P* < 0.05 (Table 1). Meanwhile, the hair dye use of case mothers had higher proportion than controls (71.4% vs 60%) (Table 2).

In the macrosomia study, compared to controls, the pregnant women had higher maternal age, pre-pregnant height, and weight, and a higher proportion of first parity (55.9% vs 42.9%), menstrual irregularity (27.6% vs 16.2%) and low education level (≤9 years) (53.5% vs 33.5%) (Table 1). However, no statistical significance was observed among other characteristics of selected lifestyle in this study (Table 2).

Comparison of each sub-study group, there were no significant differences of maternal menarche age, gravidity history, alcohol use before pregnancy, smoking status, progesterone use before and during pregnancy, nearby chemical factory, recent interior decoration, regularly cooking, chief fuel used for cooking, use of range hood, radiation-proof clothes and lipstick use during pregnancy (All *P* > 0.05) (Tables 1 and 2).

### Relationship of maternal irregular menstruation and abnormal birth weight

Table 3 showed the estimated associations between considered exposures variables and the risk of abnormal birth weight. Women with irregular menstruation had significantly higher odds of having LBW infants (OR = 2.84, 95%CI: 1.59–5.08, *P* < 0.001) and macrosomia (OR = 1.97, 95% CI: 1.18–3.29, *P* < 0.001), respectively compared to the pregnant women with regular menstruation. After adjusted by maternal age, education level and pre-pregnant BMI, the associations were still statistically significant (LBW: adjusted OR = 2.79, 95% CI: 1.53–5.09, *P* = 0.001. Macrosomia: adjusted OR = 1.93, 95% CI: 1.09–3.44, *P* = 0.023).

**Table 3:** Logistic regression analysis of hair dye use pre-pregnancy, irregular menstruation, parity and birth weight

	LBW				Macrosomia			
	Unadjusted OR (95% CI)	*P*	Adjusted^a^	*P*	Unadjusted OR (95% CI)	*P*	Adjusted ^b^ OR (95% CI)	*P*
			OR (95% CI)					
Hair dye use pre-pregnancy	1.67 (1.01, 2.76)	0.048	1.71(1.01, 2.92)	0.048	0.74 (0.48, 1.15)	0.180	0.75 (0.46, 1.23)	0.259
Irregular menstruation	2.84 (1.59, 5.08)	< 0.001	2.79 (1.53, 5.09)	0.001	1.97 (1.18, 3.29)	< 0.001	1.93 (1.09, 3.44)	0.023
Parity history								
First	1.43 (0.88, 2.33)	0.150	1.45 (0.83, 2.53)	0.189	0.59 (0.39, 0.91)	0.017	1.14 (0.67, 1.94)	0.643
≥ 2	1.00 (Reference)		1.00 (Reference)		1.00 (Reference)		1.00 (Reference)	

*Abbreviations*: *LBW* low birth weight, *95% CI* confidence interval, *OR* odds ratio

^a, b^Logistic regression models are adjusted for potential confounders (maternal age, maternal education level and pre-pregnant body mass index) as described in methods

### Relationship of hair dye use and abnormal birth weight

An increased risk of hair dye use associated with low birth weight was observed in LBW study (OR = 1.67, 95% CI: 1.01–2.76, adjusted OR = 1.71, 95% CI: 1.01–2.92, all *P* < 0.05). However, there were no statistical significance in macrosomia study (OR = 0.74, 95% CI: 0.48–1.15, adjusted OR = 0.75, 95% CI: 0.46–1.23, all *P* > 0.05).The confounding factors including maternal age, maternal education level and pre-pregnant body mass index were adjusted in the logistic regression analysis (Table 3).

### Joint effects on low birth weight study

When we combined irregular menstruation and hair dye use, the odds of having a LBW baby was higher than those only had one risk(OR: 4.58 vs 2.16, *P* < 0.05). Meanwhile, when combined with BMI < 18.5 kg/m^2^ and hair dye use, the odds of having a LBW infant had a little higher (OR: 2.96 vs 2.52, *P* < 0.05) (Table 4). Interestingly, even adjusted by confounders, the associations were still striking in each sub-study (Table 4).

**Table 4:** Combined effects of underweight / irregular menstruation and hair dye use pre-pregnancy on LBW study

	Unadjusted			Adjusted		
	OR (95% CI)	*P*	*P*_trend_	OR (95% CI)	*P*	*P*_trend_
Type-1 model ^a^						
Neither of them	1.00 (Reference)			1.00 (Reference)		
Only BMI < 18.5 kg/m^2^ or hair dye use pre-pregnancy	2.52 (1.33, 4.78)	0.005	0.012	2.53 (1.30, 4.93)	0.006	0.015
Both BMI < 18.5 kg/m^2^ and hair dye use pre-pregnancy	2.96 (1.39, 6.33)	0.005		3.07 (1.38, 6.83)	0.006	
Type-2 model ^b^						
Neither of them	1.00 (Reference)			1.00 (Reference)		
Only irregular menstruation or hair dye use pre-pregnancy	2.16 (1.20, 3.89)	0.010	0.030	2.07 (1.14, 3.76)	0.018	0.050
Both irregular menstruation and hair dye use pre-pregnancy	4.58 (2.02, 10.40)	< 0.001		4.53 (1.94, 10.59)	< 0.001	

*Abbreviations*: *LBW* low birth weight, *OR* odds ratio, *95% CI* 95% confidence interval, *BMI* body mass index is measured and defined as weight (kg) / height (m)^2^

^a^Logistic regression model is adjusted for maternal age, maternal education level, parity and menstruation cycle

^b^Logistic regression model is adjusted for maternal age, maternal education level, parity and pre-pregnant body mass index

### Joint effects on macrosomia study

When we combined women with pre-pregnancy BMI ≥ 24 kg/m^2^ and irregular menstruation, the risk of having a macrosomia was much higher than those had only one factor (OR: 13.05 vs 2.12, all *P* < 0.05). After adjusted by maternal age, maternal education level, parity and pre-pregnancy hair dye use, the risk was still striking (OR _adjusted_: 13.31 vs 2.09) (Table 5).

**Table 5:** Combined effect of overweight and irregular menstruation on macrosomia study

	Unadjusted			Adjusted ^a^		
	OR (95% CI)	*P*-Value	*P*_trend_	OR (95% CI)	*P*-Value	*P*_trend_
Neither of them	1.00 (Reference)			1.00 (Reference)		
Only BMI ≥ 24 kg/m^2^ or irregular menstruation	2.12 (1.32, 3.43)	0.002	0.001	2.09 (1.26, 3.45)	0.004	0.001
Both BMI ≥ 24 kg/m^2^ and irregular menstruation	13.05 (3.64, 46.80)	< 0.001		13.31 (3.54, 50.11)	< 0.001	

*Abbreviations*: *OR* odds ratio, *95% CI* 95% confidence interval, *BMI* body mass index is measured and defined as weight (kg) / height (m)^2^

^a^Logistic regression model is adjusted for maternal age, maternal education level, parity and hair dye use

## Discussion

To our knowledge, this is the first nested case-control study to report the association of combined pre-pregnancy hair dye use and irregular menstruation with abnormal birth weight in China. In this study, we found that women who used hair dye pre-pregnancy had significantly high risk of low birth weight infants. Simultaneously, women with irregular menstruation had significantly high risk of low birth weight infants and macrosomia. Then, when we combined the hair dye use pre-pregnancy and irregular menstruation, we found the risk of low birth weight infants were higher than those with only one risk factor. Our results showed that women with exposure of hair dye pre-pregnancy and irregular menstruation had an increased risk of low birth weight infant.

As society develops, hair dye using is rapid increasing and becoming common during women. Recent studies focused on the effects of occupational exposure to chemical substances which persist in hair care products on abnormal birth outcome and maternal complications in hairdressers [20–22, 26–29] and compared with salesclerks[21, 30–32], but the results are inconsistent. A meta-analysis showed hairdressers and cosmetologists had a significant increased risk of neonatal complications, such as LBW, fetal loss, SGA (small for gestational age) [33, 34]. Our results showed women customers who used hair dye pre-pregnancy had high risk of LBW (OR _adjusted_ = 1.71, 95% CI: 1.01–2.92).

The use of hair dye is popular between women however different components of chemical substance can be found on it. A Canadian study found the hair dye production contained at least one phthalate at a detectable level [35]. Based on the results of the epidemiological studies examined, we therefore hypothesized that the products of hair dye contains chemical substances such as ultraviolet filters, phthalate esters and parabens, et al., which suspected to endocrine disrupters [36, 37]. Endocrine disorder of women may influence the secretion of progestational hormone which could increase the risk of LBW. Also, different hair dye with less chemical products could have lower influence on LBW.

Irregular menstruation may be the other risk factor for abnormal birth weight infant. Our study reported that women with irregular menstruation had significant association of LBW (OR = 2.84, 95% CI: 1.59–5.08) and macrosomia (OR = 1.97, 95% CI: 1.18–3.29). The results were consistence with previous studies [23, 38-41], but conflict with the study of Wang ET [42], which reported that obese women with irregular menstruation had significantly risk of lower birth weight compared with regular subjects. Inconsistent results may be due to different menstrual cycle, different sample size, study population and different study design etc. Besides of the conflictive results, our results were in line with most of previous studies.

Irregular menstruation may indicate ovulation disturbances which associated with sub-fertility. Women with sub-fertility were spontaneously considered to have a risk of abnormal birth outcomes, such as preeclampsia, low birth weight, preterm birth, abortion and stillbirth [43]. Dysfunction of the endocrine system could affect concentration of serum insulin-like growth factor-1(ILGH-1), which associated with infant birth weight [44–47]. Besides, the concentrations dynamically changed during different stages, which increased in the early pubertal stages and then slowly decreased in late stages [48]. In addition, Xu et al. [49] speculated that the physiology effect of ILGH-1 on fetal growth could be a possible link between abnormal birth weight and maternal irregular menstruation.

Interestingly, when we combined hair dye use with irregular menstruation, the risk of LBW was significantly higher than those had only one risk (OR: 4.58 vs 2.16), which indicated that two factors simultaneously existed would highly strengthen the risk of LBW. As we knew, this is the first report about the joint effect of hair dye use and irregular menstruation on low birth weight infant. A Japanese study showed that the frequently use of hair dye was associated with an increased risk of abnormal circulating testosterone levels [50], which could affect the concentration of serum insulin-like growth factor - 1(ILGH-1) [44–47]. And then, Xu et al. recognized irregular menstruation also affect the secretion of ILGH-1 [49]. When these two risk factors simultaneously existed, the concentration of ILGH-1 would have greatly change, and the effect on the abnormal birth weight would be much stronger. This is a significant discovery for antenatal and newborn care.

Pre-pregnancy BMI may be other risk factors of abnormal birth weight infants. Previous studies showed that women either underweight or overweight and obese had unhealthy impacts on birth weight [51–54].Our study showed that women who used hair dye with BMI < 18.5 kg/m^2^ had higher risk of low birth weight infants than those had one factor (OR: 3.07 vs 2.53, *P*_trend_ = 0.015), irregular menstruation women with BMI ≥ 24 kg/m^2^ had higher risk of macrosomia than one factor existed (OR: 13.31 vs 2.09, *P*_trend_ = 0.001). This indicated that women with abnormal pre-pregnancy BMI with irregular menstruation or hair dye use could further strengthen the risk of abnormal birth weight infants.

The advantage of this study was the prospective design in a large sample aimed to investigate the relationship of hair dye use or irregular menstruation with infant birth weight. In addition, there were several limitations in our study. First, the information of irregular menstruation was collected by women self-reported, retrospective bias was inevitable. In future study, we could consider combine ultrasound examination-based gestational age and self-reported. Second, the detailed information of pre-pregnancy hair dye use, including the frequency and name or types of products, were not collected, which limited the quantity study of hair dye use with abnormal birth weight. This will be considered in future studies. Third, our nested case-control study did not have a large number of subjects even though the birth cohort study sample was a good number. In addition, we will do further studies because the evidences of reporting the risk of pre-pregnancy hair dye use or irregular menstruation with abnormal birth weight are limited at present.

## Conclusions

From this nested case-control study, we found that either pre-pregnancy hair dye use or irregular menstruation was associated with abnormal birth weight in China, indicating that pre-pregnancy exposure to hair dye and irregular menstruation may be risk factors for abnormal birth weight infants.

## Abbreviations

BMI: Body mass index
CI: Confidence interval
ILGH-1: Insulin-like growth factor-1
LBW: Low birth weight
NBW: New birth weight
OR: Odds ratio
SD: Standard deviation
